# Creep Behaviour of Recycled Poly(ethylene) Terephthalate Non-Woven Geotextiles

**DOI:** 10.3390/polym13050752

**Published:** 2021-02-28

**Authors:** Mateus Porto Fleury, Lucas Deroide do Nascimento, Clever Aparecido Valentin, Jefferson Lins da Silva, Marta Pereira da Luz

**Affiliations:** 1 São Carlos School of Engineering (EESC), University of São Paulo (USP), São Carlos 13566-590, Brazil; lucasdn6@gmail.com (L.D.d.N.); cclever@sc.usp.br (C.A.V.); jefferson@sc.usp.br (J.L.d.S.); 2 Eletrobras, Furnas Centrais Elétricas S.A., Aparecida de Goiânia 74923-650, Brazil; martaluz@furnas.com.br; 3 Industrial and Systems Engineering Postgraduate Program (MEPROS), Pontifical Catholic University of Goiás (PUC Goiás), Goiânia 74605-010, Brazil

**Keywords:** creep behaviour, unconfined creep tests, accelerated creep tests, non-woven needle-punched geotextiles, recycled poly(ethylene) terephthalate

## Abstract

At the beginning of this century, due to well-established Brazilian recycling processes, geosynthetics’ manufacturers started to use recycled poly(ethylene) terephthalate (PET) yarns/filaments (from PET bottles) in geotextile production. Despite the fact that recycled products cannot act as reinforcement functions, geosynthetics are constantly under sustained tensile load and experiences evolutions of the axial strain (creep behaviour). Thus, this study aims to assess the influence of the structure of (needle-punched) non-woven geotextiles manufactured using recycled PET yarns on their creep behaviour. Two geotextiles with different fibre/filament production processes were investigated (short-staple fibres—GTXnwS—and continuous filaments—GTXnwC). Unconfined in-isolated conventional and accelerated (using the stepped isothermal method) creep tests were performed at 5%, 10%, 20%, 40% and 60% of geotextiles’ ultimate tensile strength. The geotextiles investigated provided similar creep behaviour to geotextiles manufactured with virgin PET material. The standard deviation of the axial strain tends to increase as the load level applied increase. The structure of the GTXnwS harms its tensile –strain behaviour, promoting axial deformation under sustained loads, at least 50% higher than GTXnwC for the same load level applied. The influence of the load level and geotextile structure in the initial axial strain is pointed out. Long-term predictions based on creep tests performed using the stepped isothermal method have proven to be conservative and they must be restricted for quality control of the investigated geotextiles.

## 1. Introduction

Polymeric materials, such as geosynthetics, submitted to sustained axial tensile loads experience time-dependent elongation/strain, so-called ‘creep behaviour’, due to the polymers’ viscoelastic behaviour [1,2,3,4,5,6,7,8,9]. Geosynthetics’ creep behaviour is commonly evaluated in-isolation conditions and requires a series of creep tests performed at different load levels for a period that must exceed 1000 h and ideally achieve 10,000 h (NBR 15,226 [10]; EN ISO 13,431 [11]; ASTM D 5262-07 [12]). The results, typically plotted in a semi-logarithmic scale (axial strain vs. log time), exhibit three creep stages [13,14,15,16,17], and an almost linear relationship that can be represented by the best-fit Equation (1), where $\varepsilon_{t}$ is the creep strain at the specific time (dimensionless); $T_{\alpha }$, is the creep strain rate (represents the slope of the linear regression line; in s^−1^); t, is a specific time (in s); and b is the axial strain of the geotextile relative to the unity (dimensionless) [18]. Thus, the geosynthetics’ creep behaviour comprises an initial axial strain ($\varepsilon_{o}$) followed by its increase over time (creep strains):

(1) $$\varepsilon_{t}\;=\;T_{\alpha }\;.\log t\;+\;b$$

The creep behaviour of geosynthetic materials is mainly affected by the geosynthetics’ manufacturing process (type), the characteristics of its former polymer and the load level applied. Structured geosynthetics (with aligned filaments/yarns; i.e., geogrids and woven geotextiles) have a smaller creep tendency (that means, the tendency to develop creep strains overtime under a sustained load) than the non-structured ones (non-woven geotextiles) [2,19,20,21,22,23,24,25,26,27]. Polymers that possess glass transition temperature ($T_{g}$) smaller than the usual working temperature (such as polypropylene (PP) and polyethene (PE) exhibit creep strains higher than polymers with $T_{g}$ higher than usual working temperatures (e.g., poly(ethylene) terephthalate (PET)) [28,29,30,31,32,33,34,35,36,37]. The geosynthetics’ creep behaviour exhibits a linear stress-strain relationship for low load levels [16], a non-linear viscoelastic behaviour for high load levels [38] and, at moderate load levels, the high values of creep rate experienced after the load application continuously decreases over time [39].

Especially for the present study, one must bear in mind the temperature effects in the geosynthetics’ creep behaviour. As the test temperature rises, an increase in creep strains and a decrease in the elastic stiffness occurs [25,32,40,41,42,43,44,45,46,47,48,49,50,51]. This phenomenon results from changes in the arrangement of the polymers’ chains [22] and it is not equal for all polymers [32]. The relationship between the $T_{g}$ and the test/working temperature [8,52,53] and the polymer’s crystallinity region [54] are pointed out as the main factors which indicate the influence of the temperature on the creep behaviour of polymeric materials.

The most important impact of the temperature on the creep behaviour of polymeric materials is its use to accelerate creep tests at a reference temperature and obtain the long-term creep behaviour of the material tested [23,55]. The results obtained by creep tests performed in specimens submitted to different temperatures (at a specific sustained load level) can be horizontally shifted to provide a master curve that indicates the long-term creep behaviour of the material at a reference temperature [23,48,49,56,57,58]. This ‘time-temperature superposition’ (TTS) principle is based on the creep strain dependence on temperature [30] and the Boltzmann superposition principle. This procedure has been widely used by the industry to perform creep tests in polymers [5,8] and then started to be used in geosynthetic materials (e.g., [13,15,30,38,44,48,59,60,61,62]).

The value of the shift factor ($\alpha_{t}$) can be obtained based on the Arrhenius equation or the analytical expression developed by Williams et al. ([63], WLF equation), namely:

(2) $$\log\alpha_{t}\;=\;\frac{E}{R}\left(\frac{1}{T}-\frac{1}{T_{R}}\right)$$

(3) $$\log\alpha_{t}\;=\;\frac{-C_{1}\left(T-T_{R}\right)}{C_{2}+T-T_{R}}$$

where $\alpha_{t}$ is the shift factor (dimensionless); E, activation energy (J/mol); R, universal gas constant (J.K^−1^.mol^−1^); T, tested temperature (K); $T_{R}$, reference temperature(K); $C_{1}$(dimensionless) and $C_{2}$ (K), are constants that slightly vary accordingly with the polymer type and load level (dimensionless). Farrag [58] mentioned the difficulty to establish correct activation energy values since the strain rate constantly changes during the test and observed reasonable agreement between master curves obtained from Equation (3) and experimental results. Moreover, different geosynthetics manufactured with the same polymer can exhibit different values of activation energy [64] resulting from different creep mechanisms [65,66]. Thornton and Baker [61] observed a limitation of the shift factor obtained from Equation (3) for higher temperatures and reported non-conservative regression lines.

The master curve generated using the TTS technique is obtained by the juxtaposition of creep strains experienced by different specimens, each one subjected to different temperatures. This process suppresses the specimen-to-specimen variability and makes the master curve inaccurate [54]. Furthermore, the low creep tendency and temperature effect in PET-based geosynthetics impairs the use of the TTS technique. Bearing in mind these limitations, Thornton et al. [67] developed a particular case of the TTS technique, called ‘stepped isothermal method’ (SIM), which consists of the creep test performance under a sustained load level in a single geosynthetic specimen submitted to different isothermal steps. The creep strains obtained in each isothermal step are rescaled to provide a master creep curve by selecting analytical shift factors that provide a smooth curve with no creep strain superposition and sharp changes in the creep rate. A detailed explanation for scaling and shifting SIM data was reported by Zornberg et al. [37], Thornton et al. [67], Bueno et al. [68] and ASTM D 6992-16 [69].

Creep tests using SIM are indicated as a quality control method for products that have well-established creep behaviour (obtained from the conventional creep test; ISO 20,432 [70]). However, predictions of creep strains with SIM compare very well with creep strain obtained with conventional creep tests for PP geotextiles, PET non-woven geotextiles [28,68], PET geogrids [14,54,60,66] and high-density polyethylene (HDPE) geogrids [32,66]. Differences are mainly in the initial axial strain and can be a result of the load system adopted, test equipment and specimen-to-specimen variability. Bueno et al. [68] observed similar values of creep strain rates ($T_{\alpha }$ in Equation (1)) for duplicated specimens and highlighted that accelerated creep tests using SIM provide reasonable creep prediction considering the structural and polymer creep. Moreover, Bueno [71] and Thomas and Nelson [72] proved that accelerated tests using the SIM can be seen as a valuable alternative to predict the long-term creep behaviour of geosynthetics and provide valuable information for designers.

The Brazilian expertise in PET bottles’ recycling processes provides recycled materials (yarns) with high molecular weight. According to NBR 16757-1 [73], geosynthetics manufactured with these recycled materials can only be applied for environmental solutions since they do not exhibit reinforcement function and meet the specified intrinsic viscosity requirements. However, non-woven geotextiles are versatile materials used for several functions in civil and environmental engineering. Regardless of its application, it is continuously under sustained tensile load that culminates in excessive axial deformation. Considering that non-woven geotextiles fail primarily by the excessive deformation [74] and its properties are modified when they are loaded (tensile) [75], additional studies are required to address and understand the behaviour of non-woven geotextiles under sustained axial load, that means, under creep behaviour.

Furthermore, the study of non-woven geotextiles with recycled PET yarns is still incipient, and little information are present in the literature (especially considering its creep behaviour). Since the non-woven geotextiles’ structural characteristic dominate its axial deformation [2], this study aims to assess the influence of the structure of (needle-punched) non-woven geotextiles manufactured using recycled PET yarns on their creep behaviour. The geotextiles were obtained by different fibre or filament production processes (manufacturers). This study also explores the difference in the creep behaviour obtained by the conventional and accelerated creep tests for both materials investigated.

## 2. Materials and Methods

### 2.1. Characteristics of the Geotextiles

This study evaluated the performance of two needle-punched non-woven geotextiles manufactured with recycled poly(ethylene) terephthalate (PET) yarns. The recycled PET yarns adopted by the Brazilian manufacturers, initiated at the beginning of this century, results from the PET bottle recycling process commonly used as liquid containers by the industry (raw PET filaments are partially adopted when the PET bottle waste reduces). The first one is manufactured within continuous filaments (GTXnwC) and the other one within the short-staple fibres (GTXnwS). The difference in the fibre production process identifies the difference in the geotextile creep behaviour related to its manufacturing process.

The manufacturers supplied each geotextile in 5.0 m long rolls (in the transversal direction). The roll’s lateral edges (300 mm) were discarded. Geotextile specimens were obtained to perform characterization and creep tests. The creep tests’ specimens were obtained randomly in the roll’s longitudinal direction and are 500 mm × 200 mm (longitudinal and transversal direction, respectively).

The properties of both geotextiles used in this study, the relevant standard testing following the Brazilian Association of Technical Standards [76,77] and the American Society for Testing and Materials [78,79,80,81], the number of specimens tested, the mean value and the coefficient of variation (COV) are summarised in Table 1. Even within an industrial manufacturing process, non-woven geotextiles have a higher variability in their characteristics due to a heterogeneous configuration resulting in high COV values observed from each property. Considerable variability in geotextile creep behaviour is expected.

### 2.2. Creep Test Program

The test program consists of assessing the creep behaviour (or long-term response) of specimens obtained from two geotextiles (GTXnwC and GTXnwS) using an in-isolate (unconfined) creep test in conventional and accelerated conditions. Unconfined in-isolation creep tests (ASTM D 5262-07 [12]), henceforth called “conventional creep tests”, and accelerated creep tests using the stepped isothermal method (SIM; ASTM D 6992-16 [69]) were performed in 200 mm wide per 500 mm long specimens, on the specimens’ machine direction (MD). The tests were performed at load levels corresponding to 5%, 10%, 20%, 40% and 60% of the mean value of geotextiles’ ultimate tensile strength ($T_{ult}$; obtained accordingly with ASTM D 4595-05 [80] at room temperature; Table 1) for both conventional and accelerated creep tests.

**Figure 1:**
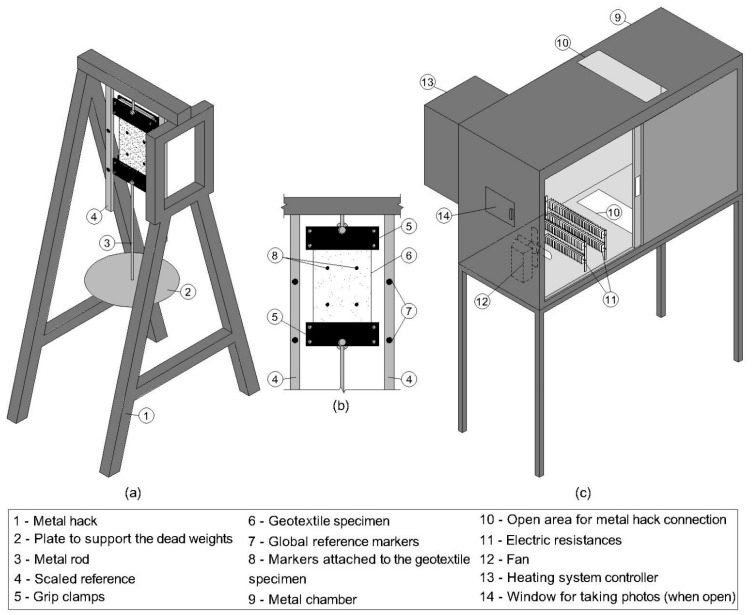
Schematic view of the (**a**) equipment to perform in-isolation (unconfined) creep tests, (**b**) close-up view of the geotextile specimen and reference markers to obtain the specimen’s strain, and (**c**) temperature-controlled chamber constructed to perform the accelerated creep tests.

For the conventional creep test, three specimens were tested for each load level applied in the metal hack illustrated in Figure 1a at the room temperature of (25.0 ± 1.0) °C. The loading system consists of dead weights applied at the bottom grip clamp of the device since it is considered a precise and stable method to apply creep loads [30]. The vertical elongations were measured through close-range photogrammetry using pictures taken at specific times throughout the conventional test (1, 2, 4, 8, 15, 30, 60 min, 2, 4, 8 h, 1, 3, 7, 14, 21 and 42 days).

The close-range photogrammetry method adopted in this study is based on the Bueno [82] photographic technique. The geotextile specimens were arranged in the clamp device with four markers attached and close to a metal scale with another four reference markers (Figure 1b). Before loading, the distance between the pair of markers attached to the geotextile and each reference marker were measured physically (in the equipment) and by a picture taken to determine the transformation constant from the local to the global system. Having defined the transformation constant, the difference between the elongations of the markers attached to the geotextile specimens in prescribed times relative to their initial position provides the specimen strain. This method has a resolution of 0.05 mm and was also used by Baras et al. [28], Bueno et al. [68] and França et al. [83]. Despite this method providing a longitudinal and lateral elongation of the geotextile specimen, only the longitudinal strains were investigated in this study. The necking caused by the specimens’ lateral contraction under a sustained load condition was evaluated by Bueno et al. [68] and is not within the scope of this study.

To perform the accelerated creep test, a temperature-controlled chamber was constructed (Figure 1c) and attached to the metal hack used in the conventional creep tests (Figure 1a). The chamber is similar to the one used by Baras et al. [28] and Bueno et al. [68], but it was constructed with a metal structure (rather than wood). The heating system consists of electric resistances and fans to achieve a uniform temperature of the system (specimen and chamber) with ± 0.5 °C of accuracy. Temperature jumps of 15 °C were achieved in 60 s. The heat system controller was positioned in the side panel of the equipment (Figure 1c). Thermocouples located in contact with the geotextiles’ specimens were used to assess the specimens’ temperature. The geotextile specimens (200 mm wide per 500 mm long) were arranged in the structure in its side panel and the photos (used to strain measurements) were taken from a window positioned in the front part (Figure 1b). The loading system is similar to those used in the conventional test, comprising dead weights connected to the grip clamp through a metal rod.

In the abovementioned equipment, accelerated creep tests were conducted using four isothermal steps (two hours long per step). The test was initiated at room temperature of (25.0 ± 1.0) °C and, after two hours from the first isothermal step, a first temperature jump of 15 °C was achieved in 60 s. Another three two-hour long isothermal steps were performed at the temperatures of 40, 50 and 60 °C within temperature jumps of 10 °C (achieved in 60 s). This test plan is similar to those adopted by Jeon et al. [15], Baras et al. [28], Zornberg et al. [37], Hsuan and Yeo [32], Thornton and Backer [61] and Bueno et al. [68]. The longitudinal elongations were obtained for 1, 2, 4, 8, 15, 30, 60 min and 2 h through the same photogrammetry method used in the conventional creep test. Due to the high coefficient of variation of the geotextile index properties (Table 1), four specimens of each geotextile (GTXnwC and GTXnwS) were tested for each load level applied in the accelerated creep tests. In this paper, the creep behaviour of the specimens was expressed in terms of the evolution of creep strains over time (in seconds).

## 3. Results

**Figure 2:**
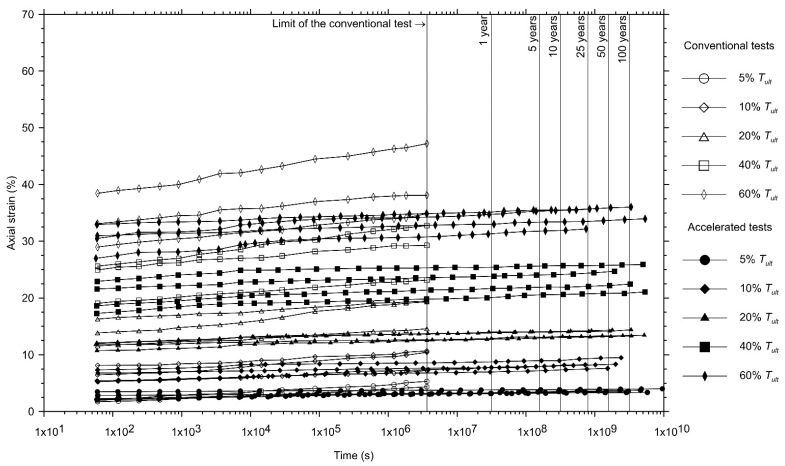
Comparison between the creep curves obtained using conventional creep tests and the master curves obtained by the accelerated creep tests for the non-woven needle-punched geotextile manufactured with continuous filament yarns (GTXnwC).

**Figure 3:**
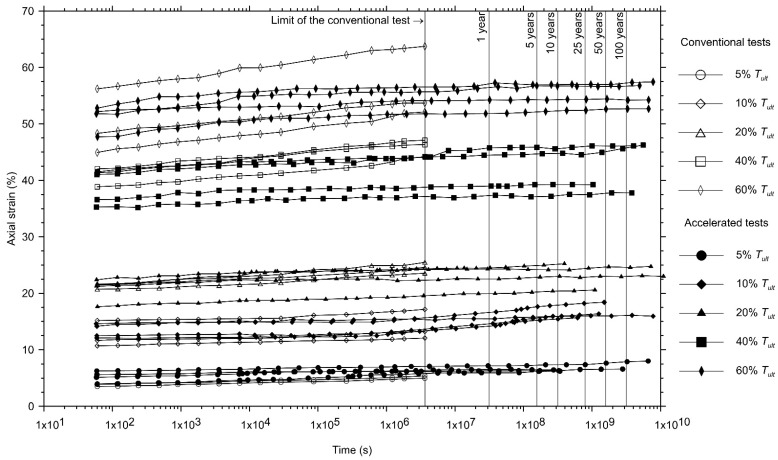
Comparison between the creep curves obtained using conventional creep tests and the master curves obtained by accelerated creep tests for the non-woven needle-punched geotextile manufactured with short-staple fibres (GTXnwS).

Figure 2 and Figure 3 show the creep curves on the semi-logarithmic scale of the non-woven needle-punched geotextiles manufactured with continuous filament (GTXnwC) and with short-staple fibres (GTXnwS), in this order. The figures present the creep curves obtained from the conventional creep test (1000 h long—three curves for each load level applied) and the master creep curves obtained from accelerated creep tests using the stepped isothermal method (SIM—four master curves for each load level applied).

**Figure 4:**
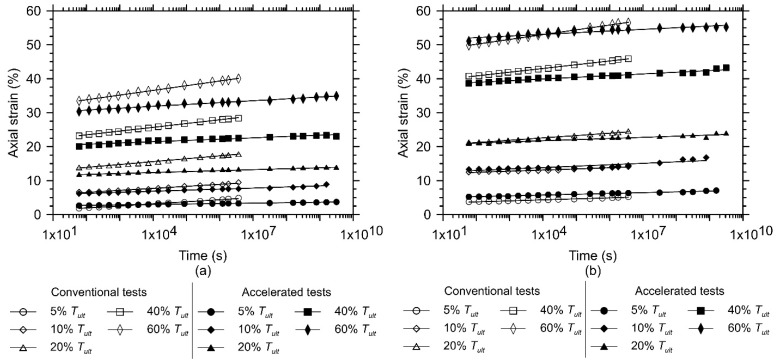
Mean creep behaviour of the non-woven needle-punched geotextile manufactured with (**a**) continuous filaments (GTXnwC) and (**b**) short-staple fibres (GTXnwS); based on three and four specimens submitted to the conventional and accelerated creep tests, respectively.

Figure 4a,b present the average creep behaviour of GTXnwC and GTXnwS, respectively, for both conventional and accelerated creep test results. Since no tertiary stage has been experienced by the geotextile specimens (regardless of the test type and load level applied), the best-fit regression line represented by Equation (1) is valid and the creep strain rate (T_α) values, parameter “b” and the coefficient of determination ($R^{2}$ are shown in Table 2 for GTXnwC and GTXnwS.

## 4. Discussion

As expected, the creep behaviour of the geotextiles, for conventional and accelerated tests, exhibits an initial axial strain ($\varepsilon_{o}$) and an increase in the axial strain over time. As shown in Figure 2 and Figure 3, the creep master curves obtained from the accelerated creep tests exhibit a reasonable relationship with the tests conducted in the conventional condition for the geotextiles investigated. These results validate the use of the test equipment constructed to perform accelerated creep tests.

**Figure 5:**
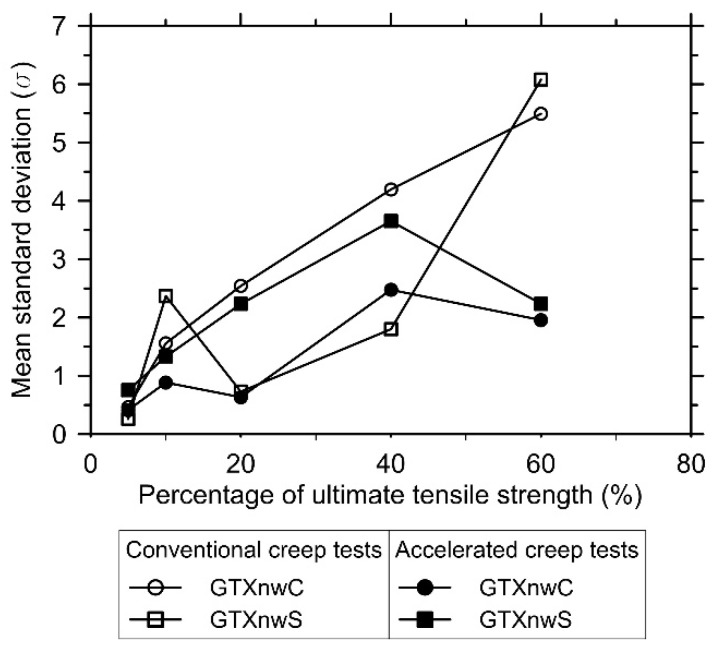
Increase in the mean standard deviation of the axial strain as a function of the load level applied in GTXnwC and GTXnwS specimens submitted to conventional and accelerated creep tests.

However, regardless of the creep test type adopted (conventional or accelerated), there is variability in the axial strain at a specific time (s). Figure 5 shows the mean value of the standard deviation (σ) as a function of the load level applied. The mean value of σ tends to increase as the load level applied increases. Both geotextiles exhibited the highest variability (standard deviation value) at the load level equal to 60% of the ultimate tensile strength ($T_{ult}$) in the conventional creep tests and equal to 40% of $T_{ult}$ in the accelerated creep tests. The smallest variability occurs at the load level of 5% of $T_{ult}$ in both test conditions (conventional and accelerated creep tests) and results from the structural elongation of the specimens, as will be explained later.

The geotextile’s creep response variability for an identical test condition (creep test type and load level applied; Figure 5) was expected since a significant variability in the geotextiles’ index test results were observed (Table 1—COV values). As the specimens’ preparation was the same for all specimens tested, the variability in the geotextiles’ creep response raises from the specimen-to-specimen variations (intrinsic variability caused by the manufacturing process) and the specimen’s response to the load application (structural elongation).

**Figure 6:**
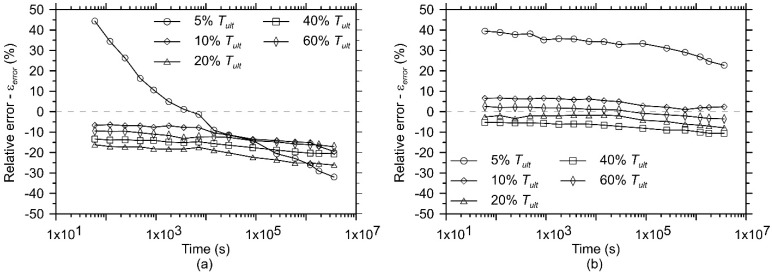
Relative strain error between the mean axial strains obtained from the accelerated creep test and the mean axial strains obtained from the conventional creep test at all load levels applied in the test program for (**a**) GTXnwC and (**b**) GTXnwS.

Despite the existence of this variability, up to the limit of the conventional test period (1000 h or 3,600,000 s), the mean creep behaviour obtained from the accelerated creep test shows reasonable agreement compared to the mean creep behaviour obtained from the conventional test (Figure 4). To compare the results obtained until the end of the conventional creep test, Figure 6 presents the relative axial strain error ($\varepsilon_{error}$) between the mean axial strains obtained from the accelerated creep test ($\varepsilon_{SIM}$) and the mean axial strains obtained from the conventional creep test ($\varepsilon_{conv.}$), calculated as follows (Equation (4)):

(4) $$\varepsilon_{error}\;=\;\frac{\varepsilon_{SIM}-\varepsilon_{conv.}}{\varepsilon_{conv.}}$$

The accelerated creep tests in GTXnwC resulted in mean axial strain values 15% lower than the ones obtained using the conventional creep test for all load levels investigated, except for the 5% of the $T_{ult}$ creep test (Figure 6a). In this latter load level, the strains measured with the accelerated creep test tend to approximate the strains measured within the conventional creep test over time, however the accelerated test overestimates the deformations after two hours of testing. This phenomenon occurs because the creep strain rate of the master curve is different from the ones obtained from the conventional creep tests. For the tests performed in the GTXnwS, the accelerated creep tests at the load level equal to 5% of $T_{ult}$ resulted in mean strain values 35% higher than the ones measured in the conventional creep test. For the other load levels investigated, there is a reasonable approximation of the strains measured with the accelerated creep tests and the conventional creep test (Figure 6b), which is better than in GTXnwC.

The mean values of axial strain obtained from the accelerated creep test show values with reasonable agreements (except for load levels equal to 5% of $T_{ult}$) in relation to the mean values of axial strain of the conventional test over the investigated period (1000 h). Thus, the assessment of the creep behaviour of the geotextiles based on the mean values of axial strains obtained from the conventional creep tests (three specimens tested) and from the accelerated creep tests (four specimens tested), as shown in Figure 4, prove to be acceptable.

Most of the average creep behaviour curves resulted in quite high values of the coefficient of determination ($R^{2}$; Table 2). The values of $R^{2}$ of the average creep curves obtained from the conventional creep test were higher than 0.98 for the load levels applied in both geotextiles (except for GTXnwS submitted to the load level equal to 10% of $T_{ult}$; $R^{2}$ = 0.95). All values of the $R^{2}$ decreased when the geotextiles were submitted to accelerated creep tests. This may be explained by the different response of each specimen to the temperature effects. With the exception of the load level of 40% of $T_{ult}$, all average creep curves exhibited values of $R^{2}$ higher than 0.97 for the GTXnwC. In the case of GTXnwS, the logarithmic best-fit curves for the load levels of 10%, 20% and 60% of $T_{ult}$ are poor in terms of $R^{2}$ values (lower than 0.90). This fact results from the large variability of the axial strain measured in the four specimens tested at each load level applied.

The creep strain rate ($T_{\alpha }$; rate of increase in the creep axial strain over time) is indicated as the best way to compare conventional and accelerated creep test results [84]. The creep strain rate of both geotextiles (Table 2) exhibited low values that are similar to the creep strain rate of other PET geosynthetics materials [2,24,26,52,84,85] and are lower than other PP [2,26,52,86] and PE geosynthetics [85], as well as high density polyethylene (HDPE) geogrids [30,44,86,87].

The $T_{\alpha }$ values for the accelerated creep tests (Table 2) are lower than the ones obtained from conventional creep tests for both geotextiles tested. In other words, the accelerated creep tests underestimate the axial strain of the geotextiles over time. The low values of creep strain rate in the accelerated test arise from the fact that the temperatures adopted in the test program are lower than the poly(ethylene) terephthalate (PET) glass transition temperature ($T_{g}$).

These results do not inhibit the adoption of accelerated creep test results using the stepped isothermal method. The differences related to the creep behaviour of geosynthetic specimens when submitted to conventional and accelerated creep tests must be identified and quantified to enable its consideration in design. As shown in Figure 2, Figure 3 and Figure 4, the accelerated creep test is a time-saving procedure that helps designers to identify creep behaviour at different load levels for almost a decade in a very short period (weeks).

**Figure 7:**
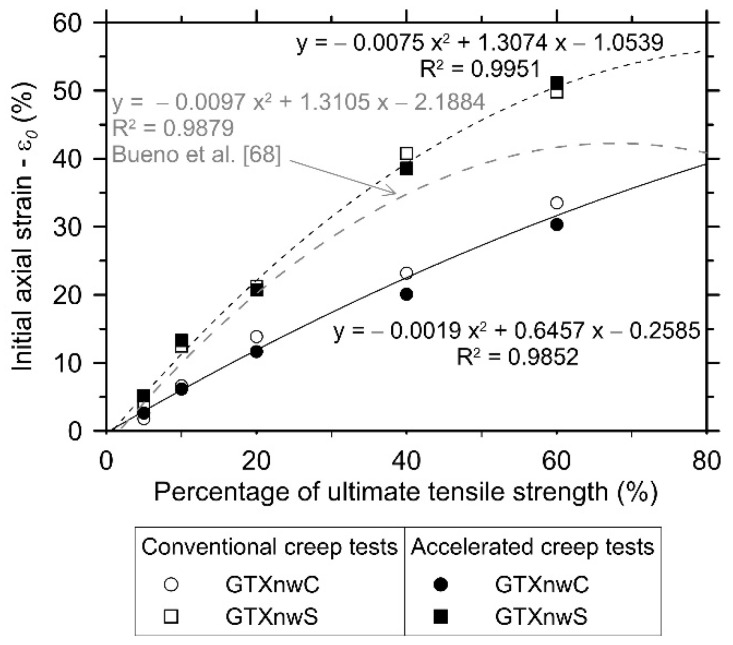
Increase in the initial axial strain as a function of the load level applied in GTXnwC and GTXnwS specimens submitted to conventional and accelerated creep tests. The same relationship is presented for the non-woven needle-punched PET geotextile tested by Bueno et al. [68].

Parameter “b” of the best-fit curves (Table 2) represents the mean axial strain of the geotextile relative to the unity (Matichard et al. [18]). The value of the initial axial strain ($\varepsilon_{o}$) reported herein is equal to the creep strain obtained 60 s after the total mobilisation of the applied load, i.e., the first reading in the test program. Figure 7 shows an increase in $\varepsilon_{o}$ as a function of the load level applied. The conventional and accelerated creep tests exhibited similar mean values of $\varepsilon_{o}$ for the GTXnwS, but a slight difference is observed for the GTXnwC, especially for the load levels equal to 20%, 40% and 60% of $T_{ult}$.

Figure 7 also presents a second order polynomial regression line for GTXnwC and GTXnwS investigated herein; and for the result of creep tests performed by Bueno et al. [68] in a similar GTXnwS. The GTXnwS investigated in this study exhibits a high increase in the mean values of the initial axial strains at the lower load levels investigated. This increase vanishes as the load level increases and it seems to provide a constant value of initial axial strain for load levels higher than 80% of $T_{ult}$. This behaviour can be associated with its highly non-uniform structure of GTXnwS. The similar behaviour observed in the results, obtained by Bueno et al. [68] (grey dashed line in Figure 7), helps to validate this non-linear behaviour. However, the abovementioned behaviour did not occur in GTXnwC. The latter exhibited almost a linear increase in the initial axial strain values as the load level increased. Further studies have to be performed at a wider range of load levels to validate this hypothesis since they were observed through extrapolation of the best-fit regression lines.

The geotextile initial axial strain ($\varepsilon_{o}$) values, when subjected to small load levels (such as 5% of $T_{ult}$), are governed by the geotextile’s structural elongation. This phenomenon occurs because the load applied does not provide the geotextile’s fully structural elongation. Hence, the axial strains developed over time have a structural elongation portion. As the load level applied increases, the portion of structural elongation mobilised (in the first minute after the full application of the load) increases. This increase is sustained up to a threshold load level applied. After reaching this point, the structural elongation is fully mobilised during the first minute and the geotextile’s axial strain over time occurs, solely, due to the polymeric properties. In this case, the strain variability is associated with the specimen-to-specimen variability in response to the load level applied. Thus, the low variability (standard deviation values; Figure 5) in the creep strains reported at 5% of $T_{ult}$ for both geotextiles results from the geotextiles’ structural deformation response to the low load level applied.

**Figure 8:**
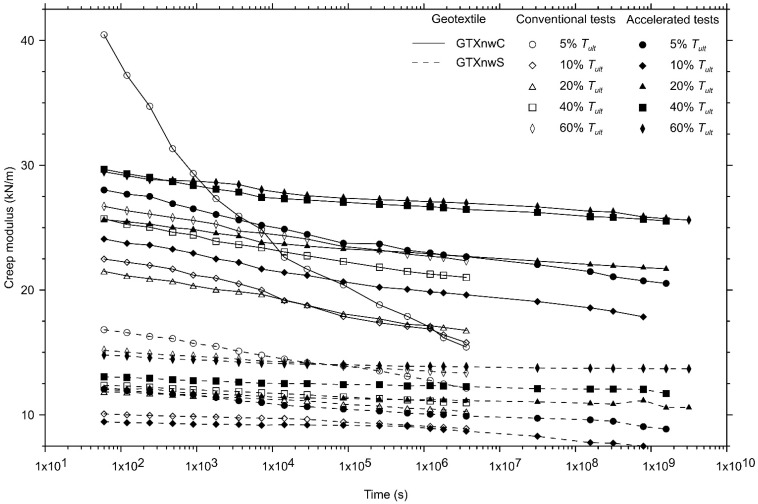
Comparison between the creep modulus obtained using conventional and accelerated creep tests for the non-woven needle-punched geotextile manufactured with continuous filaments (GTXnwC) and with short-staple fibres (GTXnwS).

The creep modulus, characterised by the ratio between the load applied and the measured axial strain (Figure 8) is the best way to characterise different types of geosynthetics [54]. As can be seen in Figure 2, Figure 3 and Figure 4, the non-woven geotextile manufactured with short-staple fibres (GTXnwS) exhibited, for all load levels, axial strains at least 50% greater than the ones experienced by the non-woven geotextile manufactured with continuous filaments (GTXnwC). Figure 8 explains this behaviour since all creep modulus curves for the GTXnwS are located below the creep modulus curves of GTXnwC.

This analysis demonstrates that the structure has a significant influence on the creep behaviour of geosynthetics—especially in the $\varepsilon_{o}$, as previously discussed. GTXnwS are manufactured with the needle-punch process in “compacted” bale staple fibres and result in a more non-uniform structure compared with the GTXnwC one. Thus, short-staple fibre geotextiles, when subjected to axial tensile load, experience a high level of structural elongation resulting from the accommodation of the fibres that induce higher $\varepsilon_{o}$ and harms the geotextile mechanical behaviour (tensile-strain relationship). The higher elongation at failure in the machine direction of the GTXnwS, reported in Table 1, supports this evidence.

Unexpectedly, the creep modulus of GTXnwS exhibited a small variation as the load level increased and the opposite behaviour was observed from the GTXnwC results. For GTXnwC, the accelerated creep test did not result in a large difference in the creep modulus compared with the results obtained from the conventional creep tests. Instead, the creep modulus for accelerated creep tests in GTXnwC resulted in values approximately 18% higher than the conventional one (except at 5% of $T_{ult}$).

The results of the test program performed in this study indicate that one may consider a high development of axial strain for the non-woven geotextiles (especially for GTXnwS) when subjected to the sustained tensile load. The lower creep modulus of the GTXnwS than GTXnwC is responsible for this excess of the creep strain developed. Moreover, GTXnwC exhibited a smaller variability in the axial strain resulting from its manufacturing process. Despite the lower values of the creep strain rate exhibited by the accelerated creep tests compared with conventional creep tests, they provide good predictions of the 1000-h creep strains of the geotextiles tested. Longer predictions have shown conservative creep strains that must be considered by the geotechnical engineers.

## 5. Conclusions

This study reported the results of a test program including a series of unconfined in-isolated creep tests performed at room temperature and unconfined in-isolated accelerated creep tests using the stepped isothermal method (SIM) conducted in two non-woven needle-punched geotextiles manufactured with recycled poly(ethylene) terephthalate (PET) yarns/filaments. The geotextiles have a different filament/yarn process and structure, one with continuous filaments (GTXnwC) and the other with short-staple fibres (GTXnwS). Based on the creep behaviour of the geotextiles tested in this study, the following conclusions are highlighted:

- The investigated non-woven geotextiles showed creep strains with similar behaviour and order of magnitude compared to other geotextiles manufactured with virgin PET yarns/filaments. The variability of the non-woven geotextiles’ creep behavior tends to increase as the applied load level increases and also stems from the structural response of each sample (specimen) to the applied load level;
- Despite the existence of this variability, up to the limit of the conventional test period (1000 h or 3,600,000 s), the mean creep behaviour obtained from the accelerated creep tests show reasonable agreement compared to the mean creep behaviour obtained from the conventional test for the load levels higher than 5% of $T_{ult}$. However, GTXnwS exhibited a more accurate prediction with the accelerated creep tests than GTXnwC;
- The representation of a mean creep behaviour of the geotextiles using three and four specimens for conventional and accelerated creep tests, respectively, provides values of the coefficient of determination ($R^{2}$) higher than 0.90 for most load levels applied (regardless of the creep test type adopted). The mean regression line indicates that the accelerated creep test underestimates the creep strains of the geotextiles investigated since it provides lower values of the creep strain rate that the conventional tests;
- A non-linear increase in the initial axial strain values as the load level applied increase was reported for both geotextiles. The smallest variability in the initial axial strain occurred at the lower load level applied (5% of $T_{ult}$). In this case, the load mobilises only a little of the structural elongation of the geotextile. For this lower load level, the creep strains developed are more governed by the specimen’s structural elongation than the filament/yarn (polymer) elongation. As the load level applied increases, the mobilised portion of the geotextile’s structural deformation increases and occurs in a shorter period, resulting in an increase in the initial axial strain; and
- The creep strains developed by GTXnwS are 50% higher (on average) than the creep strains developed by the GTXnwC. The lower creep modulus of the GTXnwS attached to the higher structural variability resulting from the manufacturing process is responsible for this significant difference in the geotextiles´ creep behaviour.

Based on the conclusions obtained from this study, one must be aware of the evolution of axial strain when non-woven needle-punched geotextiles manufactured with recycled poly(ethylene) terephthalate are under sustained tensile in geotechnical works and consider it in design. The adoption of non-woven geotextiles with continuous filaments (GTXnwC) instead of the ones with staple fibres (GTXnwS) helps to avoid problems caused by the geotextiles’ creep behaviour. Furthermore, long-term predictions based on creep tests performed using the stepped isothermal method have proven to be conservative and it must be restricted for the quality control of the investigated geotextiles.

## Figures and Tables

**Table 1:** Properties of the non-woven needle-punch geotextiles. [NOTE: The coefficients of variation (COV) are presented between parentheses in percentages].

Parameters	Testing Standard	Specimens Tested	GTXnwC ^1^	GTXnwS ^2^
Mass per unit area (g/m^2^)	NBR ISO 9864 [77]	10	269 (9.34)	384 (12.48)
Thickness (mm)	NBR ISO 9864 [77]	10	2.51 (11.53)	2.91 (9.23)
Static puncture strength (kN)	NBR ISO 12236 [76]	5	2.80 (9.16)	2.35 (16.38)
Dynamic puncture strength (kN)	ASTM D 4833-07 [78]	15	0.53 (9.65)	0.50 (18.99)
Trapezoid tearing strength MD ^3^ (kN)	ASTM D 4533-04 [81]	10	0.44 (16.55)	0.48 (13.98)
Trapezoid tearing strength CMD ^4^ (kN)	ASTM D 4533-04 [81]	10	0.46 (22.44)	0.38 (26.47)
Grab tensile strength MD ^3^ (kN)	ASTM D 4632-08 [79]	10	0.97 (15.36)	0.78 (17.01)
Grab tensile strength CMD ^4^ (kN)	ASTM D 4632-08 [79]	10	0.93 (14.02)	0.85 (15.84)
Grab breaking elongation MD ^3^ (%)	ASTM D 4632-08 [79]	10	71.32 (10.40)	87.65 (7.36)
Grab breaking elongation CMD ^4^ (%)	ASTM D 4632-08 [79]	10	77.27 (5.22)	92.47 (10.38)
Wide-width tensile strength MD ^3^ (kN/m)	ASTM D 4595-05 [80]	10	14.91 (11.35)	12.60 (14.56)
Elongation at failure MD ^3^ (%)	ASTM D 4595-05 [80]	10	48.61 (21.82)	60.1 (9.66)

Notes: ^1^, non-woven geotextile manufactured within continuous filaments; ^2^, non-woven geotextile manufactured within short-staple fibres; ^3^, machine direction; ^4^, cross machine direction.

**Table 2:** Values of creep strain rate, parameter “b” and coefficients of determination of the best-fit regression lines that represent the geotextiles’ mean creep behaviour for the conventional and accelerated creep tests performed at each load level investigated.

Geotextile	$\mathrm{Load}\;\mathrm{Level}\;(\%\;\mathrm{of}\;T_{ult})$	Creep Test Type	$T_{\alpha }{\;}^{1}$ (%/mm)	Parameter “b” (%)	$R^{2}{\;}^{2}$
GTXnwC ^3^	5	Conventional	0.6081	0.7255	0.9966
		SIM ^5^	0.1307	2.4373	0.9952
	10	Conventional	0.5913	5.3433	0.9823
		SIM ^5^	0.3150	5.6037	0.9774
	20	Conventional	0.8550	12.1600	0.9891
		SIM ^5^	0.2762	11.2789	0.9730
	40	Conventional	1.1104	21.2497	0.9985
		SIM ^5^	0.3826	19.9180	0.9370
	60	Conventional	1.3854	31.0211	0.9972
		SIM ^5^	0.5526	29.6414	0.9816
GTXnwS ^4^	5	Conventional	0.2918	4.1916	0.9871
		SIM ^5^	0.2331	5.6368	0.9813
	10	Conventional	0.3343	12.9576	0.9518
		SIM ^5^	0.4249	13.6262	0.8022
	20	Conventional	0.6908	22.2668	0.9858
		SIM ^5^	0.3241	21.6069	0.8888
	40	Conventional	1.0993	42.5398	0.9976
		SIM ^5^	0.4720	39.7159	0.9557
	60	Conventional	1.4185	52.3469	0.9981
		SIM ^5^	0.5119	52.8446	0.8961

Notes: ^1^, creep strain rate; ^2^, coefficients of determination; ^3^, non-woven geotextile manufactured within continuous filaments; ^4^, non-woven geotextile manufactured within short-staple fibres; ^5^, accelerated creep test using the stepped isothermal method.

## Data Availability

The data presented in this study are available on request from the corresponding author. The data are not publicly available since it will be used by the authors’ research group for further publications.
